# Understanding and recognition of the right ventricular function and dysfunction via a numerical study

**DOI:** 10.1038/s41598-021-82567-9

**Published:** 2021-02-12

**Authors:** Giulia Comunale, Paolo Peruzzo, Biagio Castaldi, Renato Razzolini, Giovanni Di Salvo, Massimo A. Padalino, Francesca M. Susin

**Affiliations:** 1 Cardiovascular Fluid Dynamics Laboratory HER, Department of Civil, Environmental and Architectural Engineering, University of Padova, Via Loredan 20, 35131 Padova, Italy; 2 Department of Women’s and Children’s Health, University of Padova Medical School, Padova, Italy; 3 Cardiology Unit, Department of Cardiac, Thoracic and Vascular Sciences and Public Health, University of Padova Medical School, Padova, Italy; 4 Paediatric and Congenital Cardiovascular Surgery Unit, Department of Cardio-Thoracic and Vascular Sciences and Public Health, University of Padova Medical School, Padova, Italy

**Keywords:** Biomedical engineering, Circulation, Cardiovascular diseases, Mathematics and computing

## Abstract

The role played by the right ventricular (RV) dysfunction has long been underestimated in clinical practice. Recent findings are progressively confirming that when the RV efficiency deteriorates both the right and the left circulation is (significantly) affected, but studies dedicated to a detailed description of RV hemodynamic role still lack. In response to such a gap in knowledge, this work proposes a numerical model that for the first time evaluates the effect of isolated RV dysfunction on the whole circulation. Lumped parameter modelling was applied to represent the physio-pathological hemodynamics. Different grades of impairment were simulated for three dysfunctions i.e., systolic, diastolic, and combined systolic and diastolic. Hemodynamic alterations (i.e., of blood pressure, flow, global hemodynamic parameters), arising from the dysfunctions, are calculated and analysed. Results well accord with clinical observations, showing that RV dysfunction significantly affects both the pulmonary and systemic hemodynamics. Successful verification against in vivo data proved the clinical potentiality of the model i.e., the capability of identifying the degree of RV impairment for given hemodynamic conditions. This study aims at contributing to the improvement of RV dysfunction recognition and treatment, and to the development of tools for the clinical management of pathologies involving the right heart.

## Introduction

Left ventricular systolic and diastolic dysfunctions have been studied extensively and are described as main causes of heart failure (HF)^1–3^, whereas less attention has been paid to systolic and diastolic dysfunctions of the right ventricle (RV). In the literature, the role played by RV has been largely underestimated compared to the left ventricle (LV) and, in the past, it was wrongly believed that RV could not affect cardiac output (CO) and systemic pressures^4–6^. Nowadays the importance of RV has been recognized, and its role in the cardiac hemodynamics has been highlighted^7,8^. Actually, the circulatory system is a closed-loop where the function of both ventricles is crucial. Therefore, not infrequently, impairment of one of the two ventricles precipitates impairment of the other^9^. Although LV dysfunction is most common, a recent study reported that RV myocardial infarction is not an infrequent problem, with an estimated occurrence of about 20–50% among patients with inferior myocardial infarction^10^. Unfortunately, the limited information about RV dysfunction effects does not allow to easily distinguish RV from LV dysfunctions, whereas it is evident that early diagnosis of RV myocardial infarction reduces the risk of death, cardiogenic shock, ventricular tachycardia and fibrillation, and atrioventricular block^10^.

In the present study, we implemented a lumped parameter model of the complete circulation in order to analyse for the first time the consequences of *isolated* RV dysfunction on global cardiovascular conditions. We are aimed at highlighting to what extent RV (dys)functional properties impact blood pressures and flows, so that the understanding of the “forgotten ventricle”^7^ physiopathology improves, and an easier clinical management of ventricular dysfunction will be gained in the future. The paper is structured as follows. In “Methods” section we introduce the numerical model and describe the simulations performed to analyse the different forms of right ventricular dysfunction. In “Results” section we report the results of the numerical analysis. First, we focus on flow and pressure waveforms in specific districts of the cardiovascular system, as well as on some global indexes related to the performance of the ventricles. Second, we setup the model in order to compare the outputs with clinical results of patients affected by right ventricular dysfunctions. In Sect. 4 some comments to the results are drawn and the limitations of the model are considered. Finally, conclusions on the present problem are reported in Sect. 5.

## Methods

In order to carry out an extensive analysis of different degrees of right ventricular dysfunction, we developed a closed-loop lumped parameter (0D) model able to reproduce the entire cardiovascular system. This choice represents the best cost/benefit option since 0D models provide reliable results in terms of pressure and flow waves with low computational cost^11–14^. Moreover, when the closed—rather than the open-loop configuration is adopted, 0D models fully couple the simulated state, either healthy or pathological, and the resulting hemodynamics, with no need to prescribe arbitrary flow or pressure behaviour^15^.

### Hemodynamic model

**Figure 1:**
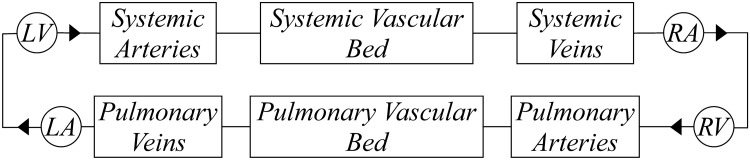
Circulation model composed of: left ventricle (LV), systemic arteries, vascular bed, and veins, right ventricle (RV), right atrium (RA), pulmonary arteries, bed, and veins, left atrium (LA) and heart valves (▸).

The circulation was simulated considering the scheme of Fig. 1. It comprises the systemic and pulmonary circulations, the four heart chambers, and valves. To simulate the conduit vascular networks, we reproduced the small resistance to flow due to blood viscosity (*R*) and the compliance due to vessel elasticity (*C*); whereas the vascular beds were represented considering only the dissipation effects (represented by *R*). The heart was represented by the elastance model^16^, employing a time-varying elastance (*E*(*t*)) to reproduce the myocardial activity^17^. *E*(*t*) was simulated via a ‘two-Hill’ function which accounts for contractile (*g*_1_) and relaxation (*g*_2_) phases of the cardiac cycle as

1 $$ E(t) = k\left( {\frac{{g_{1} }}{{1 + g_{1} }}} \right)\left( {\frac{1}{{1 + g_{2} }}} \right) + E_{min} $$

where *k* is a factor to guarantee that $\max (E(t)) = E_{max}$, $E_{max}$ being the maximum contraction force and $E_{min}$ the ventricular stiffness.

The two functions $g_{1}$ and $g_{2}$ are defined through the following expressions

2 $$ g_{1} = \left( {\frac{{t - t_{onset} }}{{\tau_{1} }}} \right)^{{m_{1} }} $$

and

3 $$ g_{2} = \left( {\frac{{t - t_{onset} }}{{\tau_{2} }}} \right)^{{m_{2} }} $$

where $\tau$ is the relative appearance in time within the heartbeat, $t_{onset}$ is the time-shift for the atrial contraction, and $m$ a suitably coefficient that controls the elastance steepness.

From Eq. (1), the pressure within each chamber (*p*(*t*)) reads

4 $$ p(t) = E(t)(V(t) - V_{p = 0} ) $$

*V*(*t*) and $V_{p = 0}$ being the chamber volume and the unstressed volume at zero pressure, respectively.

Last, the presence of the valves was considered with linear resistance effects when they are open. Notice that valves leaflets dynamics was assumed as instantaneous i.e., the “on/off diodes” model was adopted since normo-functioning valves were considered^12^. Detailed equations of all the elements that compose the lumped model are reported in the Supplementary Information.

The resulting system of ODE equations was run exploiting the built-in MATLAB function *ode15s*, solving a closed-loop system up to convergence i.e., when the relative difference between the initial values of the variables at two subsequent heartbeats was lower than 1e−4. Such a condition was usually attained within 30 cycles, requiring about 1 min of computational time with a high-performance desktop PC. Results were extracted after reaching the periodic steady state.

### Healthy case

First, the model was set up to reproduce the physiological hemodynamics of a representative healthy person.

#### Numerical simulation

The model of Fig. 1 was calibrated to meet the hemodynamics of an average adult of 75 kg and body surface area (BSA) of 1.9 m^2^, having an heart rate (HR) of 75 bpm, a cardiac output (CO) of 6.5 L/min, and a perfusion pressure of 97 mmHg^17^. Moreover, a sensitivity analysis to verify the influence of parameters on the model was carried out (as reported in the Supplementary Information).

#### Parameters’ values

The methodology proposed in Comunale et al.^18^, which is based on literature data and relationships representing physiological conditions, was adopted to properly calibrate the model according to physiological hemodynamics. For the systemic circulation, systemic vascular resistance (*SVR*) was computed as $SVR = P_{perf} /CO$, where $P_{perf}$ is perfusion pressure i.e., the mean pressure that perfuses the systemic organs. The systemic vascular resistance has three components: the arterial, venous, and vascular bed resistances. The arterial ($R_{art}$) and venous ($R_{ven}$) resistances represent the flow resistance in the large vessels, and they are $5\% SVR$ and $3\% SVR$, respectively. The larger dissipation effect was considered in the small vessels simulated by the vascular bed resistance ($R_{vb}$) which is $92\% SVR$. The arterial compliance was then computed as $C_{art} = \tau /R_{vb}$, with $\tau$ the time constant of the circulation ($\tau = 0.81 \;{\text{s}}$^19^), and the venous compliance as $C_{ven} = 30 \cdot C_{art}$^20^. For the pulmonary circulation, the value of the pulmonary vascular resistance (*PVR*) was taken from the literature^21^ ($PVR = 0.06 \;{\text{mmHg}} \cdot {\text{s/mL}}$), and we assumed the same distribution of the systemic circulation among pulmonary arterial, venous and vascular bed resistances i.e., $R_{art} = 5\% PVR$, $R_{ven} = 3\% PVR$, and $R_{vb} = 92\% PVR$. Finally, the arterial and venous pulmonary compliances were taken from Tanaka et al.^22^ ($C_{pua} = 6.7\; {\text{mL/mmHg}}$, $C_{puve} = 15.8\; {\text{mL/mmHg}}$). For the heart, values from Mynard et al.^17,23^ were used. Notice that for the healthy simulation adjustment of the unstressed volume ($V_{p = 0}$) of Eq. (4) was required to meet the desired hemodynamics. These values were then kept constant when simulating right ventricular dysfunction. Finally, valve parameters were set to simulate the resistance observed in the healthy condition. All the cardiovascular parameters are reported in Supplementary Table S1.

### Dysfunctional cases

Starting from the healthy configuration, we defined the alterations required to simulate the RV dysfunction.

#### Numerical simulation

The dysfunction of the ventricle is due to pathologies that can affect both/either the contractility of the myocardial fibers and then their capability to eject blood i.e., systolic dysfunction, and/or the ventricular passive mechanism of filling i.e., diastolic dysfunction. Accordingly, we analysed the global hemodynamics considering the following scenarios: (i) isolated right ventricular systolic dysfunction, (ii) isolated right ventricular diastolic dysfunction, and (iii) combined systolic and diastolic dysfunction.

As mentioned in the Introduction, few data are available to date for modelling the impairment of the right ventricle. In this work, the following assumptions were hence made:

- RV dysfunction is caused by the same types of impairments that affect LV i.e., systolic dysfunction is mainly determined by a reduction in RV contractile force whereas diastolic dysfunction is mostly due to increased ventricular stiffness. Thus, the parameters of the dysfunctional right heart were determined in analogy with those usually adopted for the LV impairment;
- secondary mechanisms due to the primary RV dysfunction e.g., adaptation mechanisms such as the variations of the systemic vascular resistance, were excluded to avoid additional mechanisms in the evaluation of the pathological hemodynamics given by a primary RV dysfunction.

Specific descriptions of the mechanisms of ventricular impairment are briefly described in the following subparagraphs.

##### Isolated systolic dysfunction

Systolic dysfunction (SD) refers to the ventricle’s inability to pump the adequate amount of blood due to impaired myocardial function, increased afterload, structural abnormalities, or the combination of the previous diseases^24^. SD is here reproduced through a reduction of the maximal contraction force, $E_{{\max_{RV} }}$, together with an increase in systolic times (controlled by $\tau_{{1_{RV} }}$ and $\tau_{{2_{RV} }}$ of Eqs. (2)–(3)). Indeed, it has been shown that the myocardial performance index (MPI) i.e., the ratio between the atrioventricular closure opening time (TCO) and the ejection time (ET), increases with systolic impairment^25,26^.

##### Isolated diastolic dysfunction

Diastolic dysfunction (DD) refers to the ventricular inability to fill its cavity due to decreased distensibility, delayed relaxation, and abnormal filling. For DD, there are three patterns of impaired filling, which represent progressively worse diastolic dysfunction^24,27^. In the present work, we focused on the most life-threatening condition (grade III of diastolic dysfunction) i.e., the restrictive filling. In this stage of DD, the ventricular wall stiffness ($E_{{min_{RV} }}$) largely increases whereas the tricuspid filling deceleration time ($m_{{2_{RV} }}$) decreases^28^.

##### Combined diastolic and systolic dysfunction

Combined diastolic and systolic dysfunction (CD) results from the combination of SD and DD, and it was reproduced by superimposing the two previous dysfunctions. The same grade of impairment *p* was adopted for both SD and DD for sake of simplicity in order to analyse the relative weight of each of them on the resulting hemodynamics.

#### Parameters’ values

Once the parameters reproducing the hemodynamics of a representative healthy person were determined, we introduced the percentage of impairment (*p*) in the range 0% (healthy condition)–100% (complete dysfunctional condition) to simulate the RV dysfunction. Particularly, we defined the value of the parameters in the presence of a complete (systolic/diastolic) dysfunctional ventricle (*p* = 100%) and we varied linearly all the parameters involved. To simulate the systolic dysfunction, $E_{{\max_{RV} }}$ was reduced to zero when the ventricular contractility is fully compromised, whereas $\tau_{{1_{RV} }}$ and $\tau_{{2_{RV} }}$ were increased from 0.215 s and 0.362 s (*p* = *0%*) to 0.430 s and 0.723 s (*p* = 100%), respectively. In the DD case, we increased $E_{{{\text{min}}_{RV} }}$ from 0.035 to 0.140 mmHg/mL and $m_{{2_{RV} }}$ from 21.9 to 87.6, similarly to other works focused on the LV dysfunction^29^. Analogously, to simulate CD, $E_{{{\text{max}}_{RV} }}$ was reduced, $\tau_{{1_{RV} }}$ and $\tau_{{2_{RV} }}$ were increased, and $E_{{{\text{min}}_{RV} }}$ and $m_{{2_{RV} }}$ were increased as defined above. It is worth noting that with the percentage of 100% the ventricle loses any contractility and/or compliance property, making the right ventricle a totally passive chamber with no pumping or suction function. This condition is rarely met in clinical practice because of its extreme severity, and it was here considered to obtain a full description of the pathophysiology.

See the Supplementary Information (section Time-varying elastance) and the Supplementary Fig. S3 for the description and the graphical representation of the time-varying elastances for the different dysfunctions.

### Clinical case

To compare model predictions and a real scenario, the in vivo investigation of Browning et al.^30^ was considered. In this study, patients are affected by pulmonary arterial hypertension (PAH) and DD i.e., they suffer from a condition that matches our definition of combined RV dysfunction since PAH is known to be associated with severe systolic dysfunction^31^. To setup the model, we identified the degree of impairment as follows. First, knowing that the systolic functionality alters ventricular performances i.e., EF^32^, we determined the systolic dysfunction required to obtain the EF_RV_ reduction reported by the authors^30^. Second, we tuned the degree of diastolic dysfunction to minimize the difference between the predicted results and measured data^30^.

### Ethical approval

This article does not contain any studies with human participants or animals performed by any of the authors.

## Results

**Figure 2:**
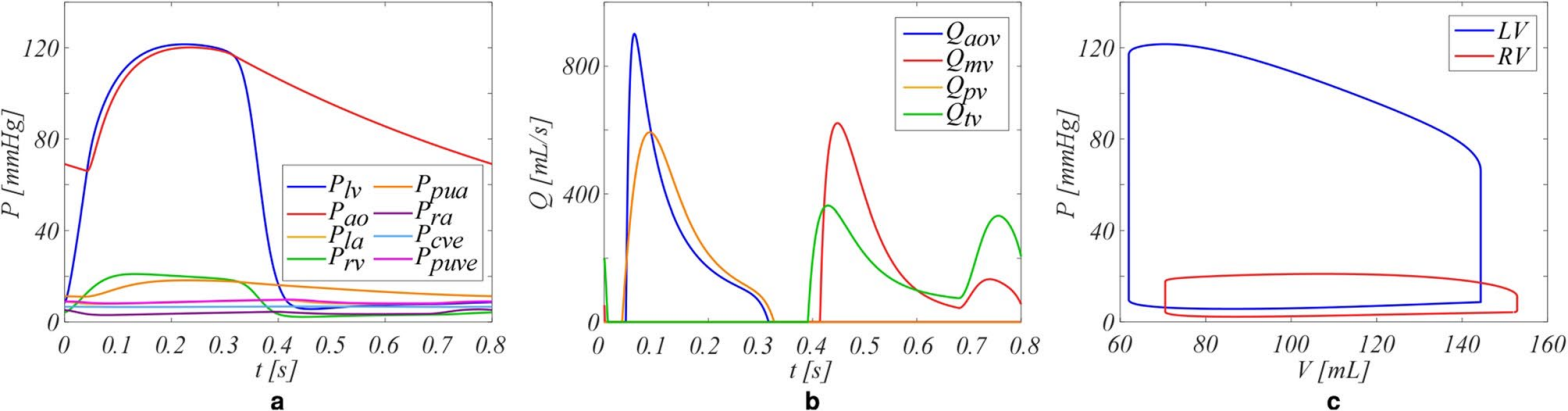
Hemodynamic waveforms of the healthy condition. (**a**) Pressures, (**b**) flows, and (**c**) pressure–volume (PV) loops. Subscripts: *lv* left ventricle, *Ao* aortic and arterial vessels, *sr* systemic vascular bed, *rv* right ventricle, *PuA* pulmonary arteries, *pr* pulmonary vascular bed, *Aov* aortic valve, *Mv* mitral valve, *Pv* pulmonary valve, *Tv* tricuspid valve. The LV presents with flow peaks which are higher than the RV, exhibiting an LV pressure almost 6 times greater than the RV estimated one, both having a stroke volume of about 80 mL.

Table 1 reports the comparison between the outputs of the healthy simulation and in vivo reference data. The model well represents the global hemodynamic variables, with both volumes and pressures within the physiological ranges. As shown in Fig. 2, the computed pressures, flows, and volumes waveforms are representative of familiar waveforms seen in these territories. The left heart is characterized by higher flow peaks than the right side, and LV exhibits a pressure almost 6 times greater than that in the RV, both having a stroke volume of about 80 mL.

**Table 1:** Comparison of the healthy condition outputs with in vivo reference data given as a mean with range.

Parameters	Units	Model	Reference
CO	[L/min]	6.2	6.5 (3.6–9.4)^21^
Total vascular resistance			
SVR	$\left[ {{\text{mmHg}} \cdot {\text{s/mL}}} \right]$	0.89	0.8 (0.5–1.1)^21^
PVR	$\left[ {{\text{mmHg}} \cdot {\text{s/mL}}} \right]$	0.06	0.06 (0.02–0.09)^21^
**Volume**			
$LV_{max}$	[mL]	144	150 (119–181)^36^
$RV_{max}$	[mL]	153	173 (134–212)^36^
$LA_{max}$	[mL]	111	97 (70–124)^36^
$RA_{max}$	[mL]	107	101 (37–177)^37^
$LV_{min}$	[mL]	62	47 (32–62)^36^
$RV_{min}$	[mL]	71	69 (47–91)^36^
$LA_{min}$	[mL]	72	44 (31–57)^36^
$RA_{min}$	[mL]	66	50 (15–92)^37^
**Pressure**			
SBP	[mmHg]	120	125 (118–132)^38^
DBP	[mmHg]	66	73 (68–78)^38^
PuA (systolic)	[mmHg]	18.3	22.5 (21.5–23.5)^39^
PuA (mean)	[mmHg]	13.5	15.5 (12.9–18.1)^39^
**E/A ratio**			
Mitral	[–]	4.6	0.6–2.6^40^
Tricuspid	[–]	1.1	0.8–2.3^40^

**Figure 3:**
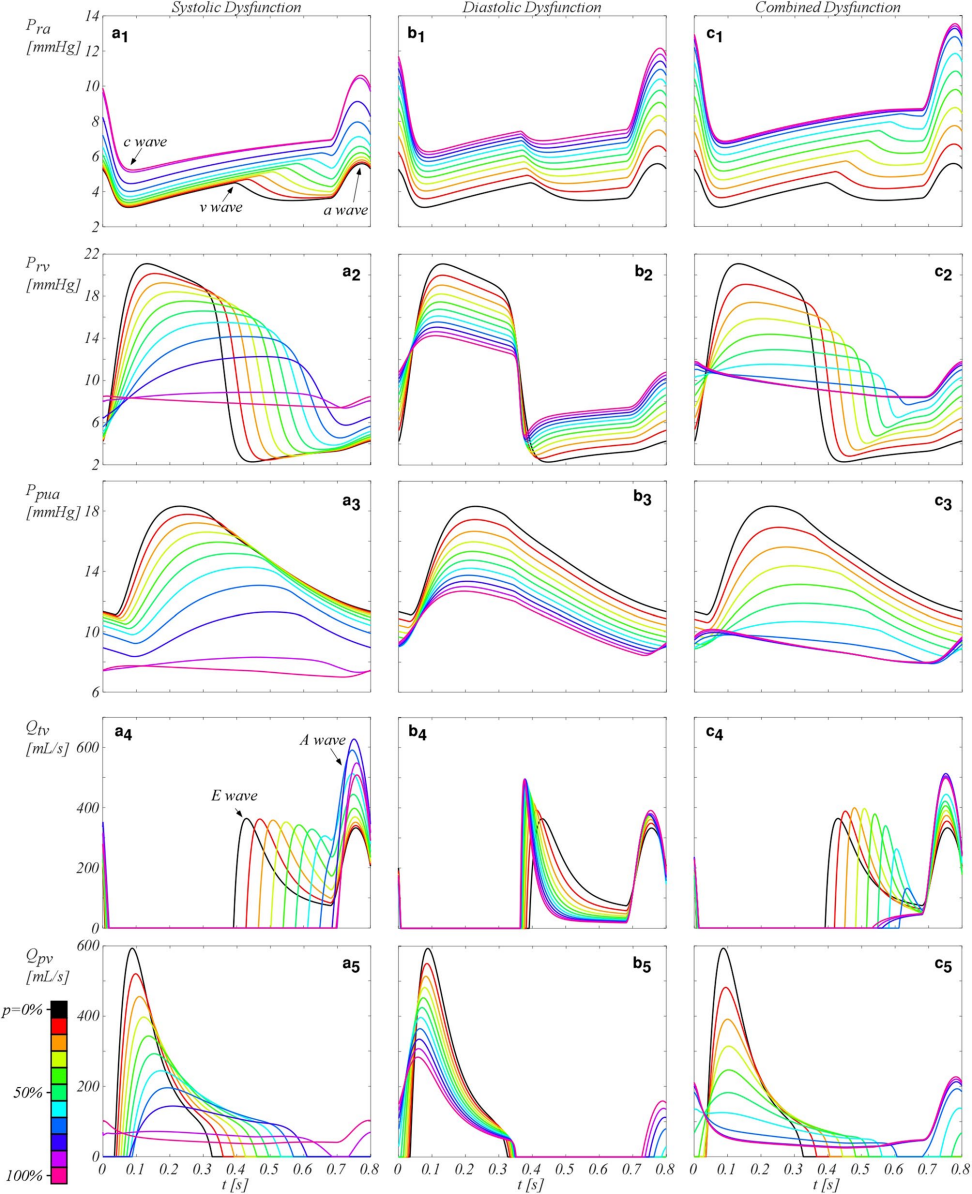
Pressures and flows for a heartbeat in the dysfunctions. (**a**) Systolic dysfunction, (**b**) diastolic dysfunction, and (**c**) combined dysfunction. P_ra_, right atrium (RA) pressure (subscript 1); P_rv_, right ventricle (RV) pressure (subscript 2); P_PuA_, pulmonary arteries (PuA) pressure (subscript 3); Q_Tv_, tricuspid valve flow (subscript 4); Q_Pv_, pulmonary valve flow (subscript 5). Black solid line, healthy configuration (*p* = 0%); coloured lines; the corresponded degree of RV impairment, *p*, from 10 to 100%. Significant values of computed pressures and flows are reported in Table S2 of the Supplementary Information.

Figure 3 shows the pressures and flows estimated in various districts of the vascular circuit for the three RV types of dysfunction. In the first column, SD outputs are presented. As *p* increases, the right atrial (RA) pressure increases. The exacerbation of the impairment also flattens the late-systolic peak (v-wave) and emphasizes the end-diastolic peak (a-wave), despite the unaltered right atrial functionality. On the other hand, RV pressure exhibits a marked reduction of the systolic peak and an increase of the diastolic pressures. On the contrary, the pulmonary arterial pressure (P_PuA_) substantially reduces on the whole heartbeat. Both the two pressures curves have flattened profiles as the pathology worsens and the systolic phase increases according to *E*(*t*) displayed in Supplementary Fig. S3a. The increase in P_ra_ is offset by the increase in the diastolic P_rv_, resulting in a reduction of the E wave. Such a reduction is partly compensated by an increase of the A wave on the tricuspid valve flow (Q_tv_) (Fig. 3a4). From an impairment of *70%* E wave completely disappears and the A wave reaches a peak of about 600 mL/s, double compared to the healthy case (300 mL/s), whose maximum is observed for *p* = 80%. The loss of contractility affects also the flow across the pulmonary valve (Q_pv_) (Fig. 3a5). Q_pv_ abruptly lessens at systolic peak and, for a complete SD, Q_pv_ shows an almost constant waveform.

The outputs due to DD are reported in the second column of Fig. 3. According to *E*(*t*) function, the shape of all the variables during the cardiac cycle remains unaltered for the different grades of *p*. The simulated pressures in the right atrium and in the pulmonary artery exhibit a positive and negative shift with *p* increasing, respectively. Consistently, P_rv_ decreases during systole and increases during diastole as DD increases. The flow between RA and RV (Q_tv_), due to their pressure variations, shows higher peaks for both the E and A waves., The former results to be characterized by an increasing flow deceleration and the latter by an increasing flow acceleration (Fig. 3b4). At the same time, the peak of Q_pv_ reduces, but the ejection time of the ventricle slightly increases. Interestingly, for severe DD (*p* > 60%), the pulmonary valve opening is anticipated at the end of diastole i.e., atrial contraction is causing pressure to rise above pulmonary arterial pressure, such that the pulmonary valve opens before the onset of ventricular systole (Fig. 3b5).

Finally, the results provided by the CD case are reported in the third column of Fig. 3. The diastolic dysfunction mostly affects the output variables during the diastole, whereas the systolic dysfunction influences the results during the whole cardiac cycle since the increment of the ejection time has an effect also in the early-diastole.

**Figure 4:**
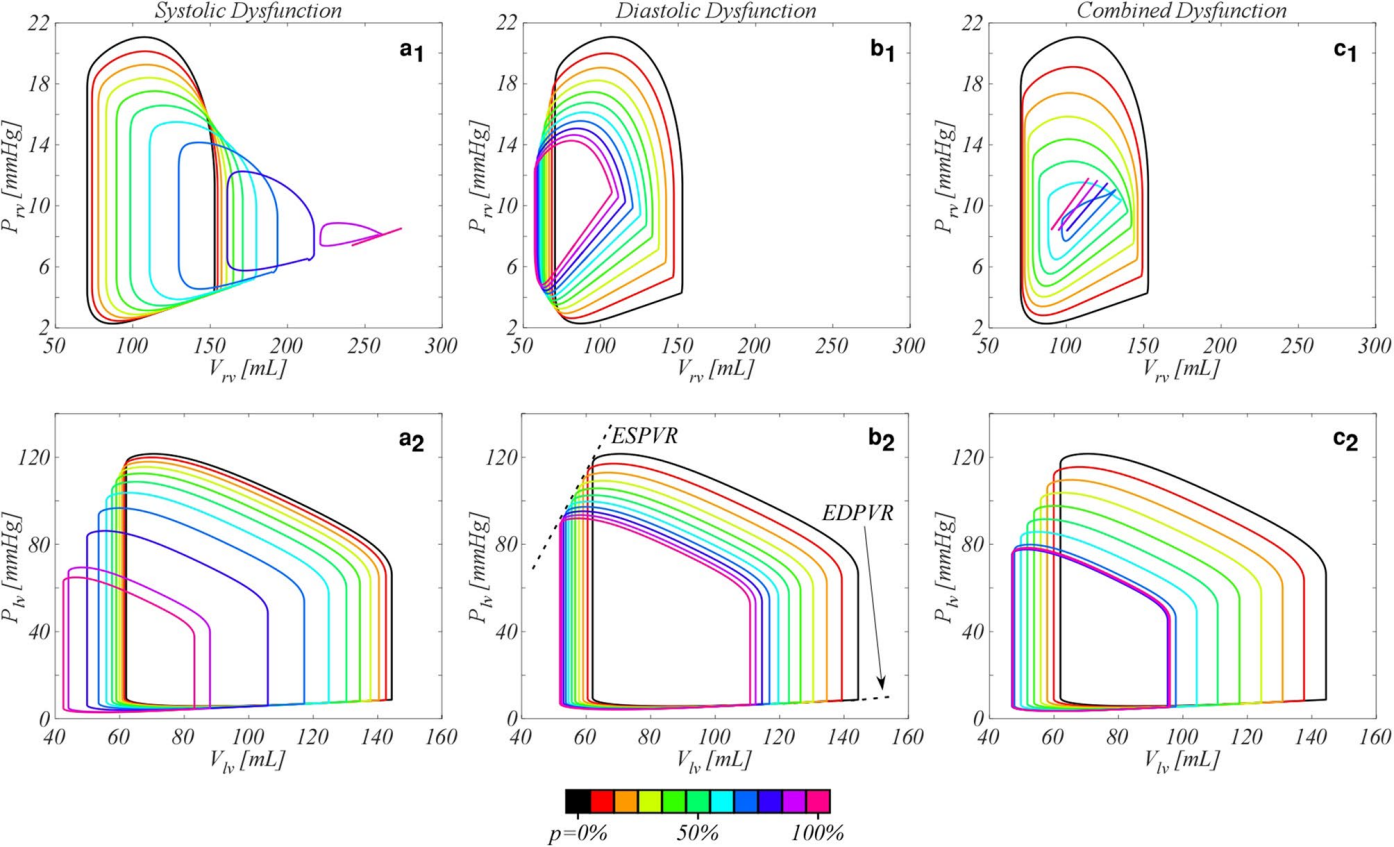
Pressure–volume loops for RV (subscript 1) and LV (subscript 2). (**a**) Systolic dysfunction; (**b**) diastolic dysfunction, and (**c**) combined systolic and diastolic dysfunction. Black solid line, healthy configuration (*p* = 0%); coloured lines; the corresponded degree of right ventricle (RV) impairment, *p*, from 10 to 100%.

Figure 4 reports the pressure–volume (PV) loops for RV (subscript 1) and LV (subscript 2) for the three dysfunctions considered. When SD is imposed on the right ventricle, RV end-systolic volume (ESV) increases and so does the RV end-diastolic volume (EDV). However, ESV increases more than EDV, resulting in a reduction of the stroke volume (SV = EDV – ESV). The impaired pumping activity causes a reduction in RV systolic pressures that, considering the volume increase, shifts the RV PV loops in downward/rightward direction. For the worst pathological condition, RV completely loses the ability to eject blood and the PV loop collapses in a line, as expected when the ventricle becomes a passive chamber (Fig. 4a1). The impairment of RV affects also LV, that shows a reduction of both the volumes and pressures (Fig. 4a2). The diastolic dysfunction has an effect on RV PV loops opposite to that due to SD (Fig. 4b1). Both RV ESV and RV EDV reduce of about 30% and 20%, respectively. The inability to fill the cavity significantly increments the end-diastolic pressures (EDPs) and at the same time reduces the systolic pressures, causing the shrinking of the loops with leftward/downward shifts. The effects on the LV are similar to the SD case, although the leftward/downward shift of the loops for *p* > 60% is lower (Fig. 4b2). In the presence of the combined dysfunction, the pressure-volumes of RV move downward such that the systolic dysfunction shift is counterbalanced by the diastolic one (Fig. 4c1). Also LV PV loops are affected by both systolic and diastolic dysfunction with PV loops in intermediate positions with respect to the SD and DD cases (Fig. 4c2).

**Figure 5:**
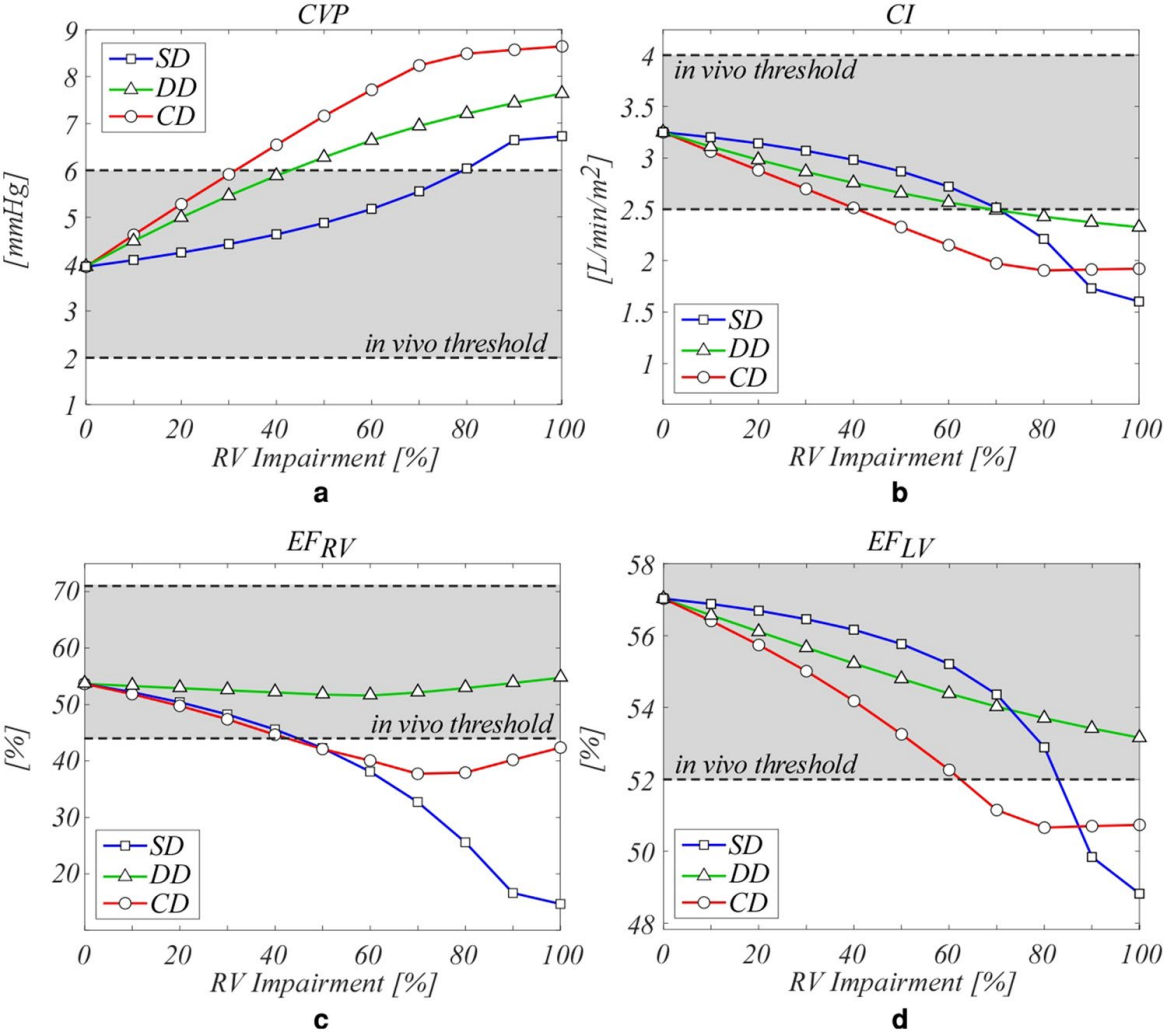
Comparison at various rates of impairment between the different types of dysfunction (symbols) and in vivo reference data (grey area) for a healthy subject. □, systolic dysfunction, ∆, diastolic dysfunction, and ○, combined systolic and diastolic dysfunction. (**a**) CVP, mean pressure in the central veins and in vivo data^53^, (**b**) CI, cardiac index and in vivo data^53^, and EF, ejection fraction for (**c**) right ventricle (RV) (EF_RV_) and in vivo data^59^, and (**d**) left ventricle (LV) (EF_LV_) and in vivo data^60^.

Figure 5 presents the mean central venous pressure (CVP), the cardiac index (defined as CI = CO/BSA), and the ejection fraction (EF) of both the ventricles. The quantities are compared to in vivo reference values, whose normal physiological ranges are indicated by grey areas. All the types of dysfunction determine an increase in CVP (Fig. 5a). SD is outside the physiological ranges for severe impairments (*p* > 80%), DD oversteps for moderate dysfunction (*p* > 40%), and CD goes beyond the ranges for *p* > 30%. All the dysfunctions depress the ventricular functionality resulting in hypoperfusion (Fig. 5b). In the early stage of impairment, SD determines a lower CI reduction than DD. For *p* > 70% CI is outside the physiological ranges for both SD and DD, however, for SD, CI plummets. On the other hand, the combination of dysfunctions speeds the overstepping of the ranges (*p* > 40%). The response in terms of EF highlights the role of RV contractility (Fig. 5c). EF_RV_ is estimated about 55% in the whole range of DD impairment. Conversely, SD causes a significant EF reduction (from 55% to 15% for *p* = 0% and *p* = 100%, respectively), and in the CD case, the loss of contractility determines an EF decrement up to *p* = 70%. For more severe CD impairment EF is almost constant. As already pointed out for the PV loops, RV dysfunction causes a diminution of LV performance for all types of dysfunction (Fig. 5d). Generally, LV results to be less compromised by the RV impairment than what RV itself is, and EF is lower than the in vivo threshold only for severe impairments in the SD and CD cases, reaching values of about 50%. On the other hand, in the DD case, EF_LV_ worsens of about 8%, remaining within physiological ranges.

Finally, in Table 2 we report the comparison between the numerical results and the in vivo data of Browning et al.^30^ for RV EDV, RV ESV, EF_RV_, and CI. The best match between clinical measurements and model prediction is found for a systolic and diastolic impairment of 67% and 20%, respectively i.e., CD with different degree of systolic and diastolic dysfunctions. In that case, the model outputs consistently estimate the indexes reported in Browning et al.^30^, with differences within the measurement error.

**Table 2:** Comparison of the CD condition outputs with in vivo reference data (mean and range) from Browning et al.^30^.

Parameters	Units	Reference	CD *p*_*systolic*_ = 67%–*p*_*diastolic*_ = 20%
$V_{{rv_{max} }}$	[mL]	181 (164–198)	172
$V_{{rv_{min} }}$	[mL]	123 (106–140)	112
$EF_{RV}$	[%]	35 (32–38)	35
$CI$	L/min/m^2^	2.5 (2.3–2.7)	2.4

## Discussion

The importance of the right ventricle has often been underestimated in the clinical practice and research^4,6,7,33,34^. A recent review even highlights that “the perception of the right ventricle has fluctuated between an essential and a nonessential ventricle”^7^. Actually, observing for example that patients with a Fontan circulation (i.e. who do not have a sub-pulmonary ventricle) survive into adulthood with quite a good life quality one might presume that RV is nonessential indeed. However, in vivo evidences are pointing out that the RV plays a key role in the onset and progression of a number of diseased conditions as well as in exercise capacity^35^. So, in order to contribute to improve the knowledge of right ventricular (dys)function effects on the complete circulation hemodynamics, we studied the *isolated* RV dysfunction by physically based numerical simulations.

We built up a lumped parameter model of both the right and left circulation that proved its capability to reproduce the physiological condition with pressures, flows, and global hemodynamic variables in a very satisfying agreement with literature data of healthy subjects^21,36–40^. In particular, hemodynamics of the right side is found to be well represented in all its characteristics, right heart flow waveforms included^41^.

A large number of information worth of discussion emerges from model simulations when RV dysfunction is taken into account. We first recall that our model includes for the first time prolonged cardiac time intervals among the mechanisms that drive the isolated systolic dysfunction (SD) condition. Indeed, the variation of systolic time with the rate of impairment, which is peculiar to the ventricular systolic dysfunction^25,26^, emerged as crucial in order to obtain results in accordance with clinical evidences. In particular, the increase in ventricular end-diastolic pressure and the reduction in the systolic one, well known for the left ventricular failure^32,42^, has been recently reported also in the case of RV systolic impairment^43^. This result is clearly represented by the model in presence of *right* systolic dysfunction, when RA is forced to increase its pressure to overcome the pressure into the overfilled ventricle and open the tricuspid valve, while P_pua_ decreases^4,44^. At the same time, the computed Q_tv_ E wave disappears due to the reduction of the tricuspid pressure gradient, in part compensated by the increase of the A wave. Notice that a similar atrioventricular valve flow is known in *left* ventricular disfunction^45^. Hence, we can speculate that it is the impairment of the right/left systolic function the pathologic condition that leads to such a right/left atrioventricular flow behaviour. We also observed that, importantly, when the contractile activity is absent (*p* = 100%), the model captures the nearly constant P_rv_ and Q_pv_. Such a result, somehow expected, shows that in absence of any contractile activity the right ventricle behaves like a passive conduit i.e., a condition similar to the spontaneous evolution of the Fontan circulation appears^46^, but with the RA still contracting.

For what the isolated diastolic dysfunction (DD) is concerned, model results suggest that when the right ventricle suffers from *restrictive filling* the ejection fraction EF_RV_ is almost preserved. In other words, the ventricle exhibits the well-known features of heart failure with preserved ejection fraction (HFpEF) i.e., of the pathology that is usually attributed to LV^34^. Such an evidence seems quite interesting since it might help in answering some of the questions left open in Gorter et al.^34^ about “the determinants of RV dysfunction in HFpEF”.While it is true that EF_RV_ is substantially maintained*,* on the other hand DD results to alter pressures and flows. In this sense, it is worth noting that model predictions for severe impairment show the appearance of a secondary peak in Q_pv_ at late diastole which, according to Gatzoulis et al.^47^ for Tetralogy of Fallot patients, could be ascribed to atrial contraction. Notice that the debate about the aetiology of such a secondary peak in Q_pv_ is still open^48^. Among possible explanations^48^, we here recall the one that ascribes the peak appearance to the atrial, ventricular, and pulmonic pressure state in *restrictive filling*, which forces the ventricle to behave like a ‘passive conduit’^47^. Indeed, since the model assumes *isolated* dysfunction and does not account for adaptive processes, the ventricular stiffness does seem to be the actual leading cause of the diastolic flow peak occurrence.

Finally, in the combined systolic and diastolic dysfunction (CD) the effects of the two previous dysfunctions sum, and both their footprint can be easily recognised in CD simulations’ results. However, the superposition of SD and DD follows a non-linear behaviour, so that CD hemodynamics exhibits an overall worst performance.

The hemodynamic alterations visible in calculated PV loops (Fig. 4) can also be commented and compared to clinical evidences. In the SD scenario the reduction of the end-systolic pressure and the increment of the volume correspond to the ventricular response reported in Monnet et al.^49^, and the computed EDPVR increase well compares to that typically occurring in diastolic dysfunction^50^. In the CD case, the right ventricle attempts to dilate for the lower contractility due to SD, but the increased passive stiffness due to DD opposes this volume dilation. For *p *$\ge$ 80%, the increased stiffness prevails so that the RV EDV reduces, resulting also in a slight increment of ventricular performance (Fig. 5b–d). Focusing on the LV PV loops, the computed shift is always downward/leftward in all the investigated cases i.e., LV work reduces. Obviously, since the left ventricle mechanical functionality is fully maintained, such a ventricular work reduction results from the entanglement between the LV and the suffering RV, which progressively drops the SV^51^. Notice also that the effect of RV systolic dysfunction on LV is more severe in the CD than in the SD case. This result is not surprising since the loss of the passive action of the right myocardium, expressed by its relaxation, worsens the ventricular working.

For what CVP and CI are concerned (Fig. 5a,b), clinical limits usually associated to potential ventricular disease^52,53^ are found to be overpassed for all the three different types of reproduced dysfunction, although for different degrees. Such an occurrence strengthens the idea that the correct functioning of the right ventricle during the whole cardiac cycle is of crucial importance in assuring overall physiological hemodynamic conditions. Passing now to EF, we observe that, as expected from PV loops’ evidences, our results confirm the strong interaction between the two ventricles. In particular, the computed EF behaviour suggests that both EF_RV_ and EF_LV_ have to be evaluated in right diseases (e.g., Tetralogy of Fallot). Indeed, van der Ven et al.^51^ recently pointed out that the role of LV in Tetralogy of Fallot can not be neglected, despite LV parameters are not considered in current guidelines for the assessment of the most appropriate timing of intervention^51^.

Finally, the comparison between the numerical simulation with the in vivo clinical data of Browning et al.^30^ (Table 2) is illustrative for the potential of the model as predictive tool to be applied to real scenarios. The model’s ability to reproduce the hemodynamic indexes seems to be particularly promising. Indeed, the differences between simulated and observed outputs are more than acceptable, for example the computed CI is 2.4 L/min/m^2^ versus the measured 2.5 L/min/m^2^. Notice that such a result strengthens the idea that the *isolated* dysfunction model effectively represents the real pathological mechanisms governing the depressed/stiffened ventricle. However, the possibility that accounting for increased pulmonary resistances further enhances model predictions of Browning et al.^30^ cases deserves attention, and will be analysed in future work.

The diagnosis and treatment of diseased conditions might hence benefit from the application of the model thanks to its ability in determining flow and pressure changes as a result of RV dysfunction. For example:

1. *Isolated right ventricular dysfunction: Uhl disease, right ventricular dysplasia, post-surgical right ventricular dysfunction.* This condition is rare, and the knowledge of heart chambers physiopathology as well as the clinical evolution assessment are hardly feasible. The present model may help to grade the correct ventricular dysfunction.
2. *Pulmonary arterial hypertension.* In this case, the pulmonary arterial pressure is augmented. The associated right heart hemodynamics can be due to the increased afterload or associated with a percentage of RV dysfunction. Distinguishing between these two cases is still a clinical challenge and a suitably implementation of the model assigning augmented pulmonary artery pressure conditions may help to identify the patients at higher risk to develop right HF.
3. *Cor pulmonare.* Left HF usually affects RV function due to the increase of left atrial pressure and the pulmonary arterial pressure. Similarly to setting (2), the RV dysfunction model, implemented with the LV impairment, may be appropriate to recognize the hemodynamics due to the left HF or associated to primitive right HF (bi-ventricular failure). This distinction is particularly important when a left ventricular assist device (VAD) is required. In the case of induced right HF, the right ventricular function recovers, partially or completely, after the left VAD implantation. Conversely, when the right ventricular dysfunction occurs, usually a bi-VAD is the best clinical option.

Finally, study limitations and possible future improvements can be summarized and commented. *Limitations*: (i) Cardiac valve flow was modelled according to a simple linear flow/pressure gradient relationship, which is likely the reason why Q_ao_ and Q_mv_ (E/A ratio) are found to have peaks somehow higher than physiological ones^40^. Moreover, the adoption of ‘on/off diodes’ as heart valves model results in the absence of reverse flow at the final stage of the open valve phase. (ii) Neither physiological compensatory mechanisms e.g., ventricular remodelling and venous constriction, nor secondary diseases e.g., tricuspid valve insufficiency^54^, were accounted for in the model since we aimed at highlighting primary RV dysfunction effects alone. *Future improvements*: (iii) The adaptation of more complex flow/pressure gradient relationships for cardiac valve flow is expected to overcome the issues highlighted at previous point (i) (Mynard et al.^23^ and Susin^55^). In particular, the inclusion of valve dynamics will allow realistic simulation of conditions affected by both RV dysfunction and diseased valve(s) (stenotic and/or incompetent). (iv) Further insights are also expected from taking into account fluid–structure interactions e.g., the remodelling of cardiac chambers as a compensation of impaired ventricular function. (v) Last but not least, further development can arise from taking into account sex differences in model parameter values and/or cardiovascular functional response i.e., differences that has been recognised as pivotal in both healthy and pathological hemodynamics^18,56–58^.

## Conclusions

Our study highlights the effect of RV (dys)function on the overall hemodynamics. The 0D circulation model seems to correctly simulate the pressures and flows in both the pulmonary and systemic circulations. Changes in RV functions lead to significant modifications of the overall hemodynamics, affecting also LV output due to a decreased preload. To the best of our knowledge, there is not such a previous engineering work aimed at understanding the hemodynamic implications of the development of RV impairment. In addition, the model matches clinical data of patients presenting RV diseases, providing a first step towards the set up and the assessment of a numerical tool able to support clinicians to improve the knowledge and the understating of the role played by RV on the blood circulation with potential future benefits in the diagnosis and treatment of RV pathologies.

## Supplementary Information

Supplementary Information.

## Data Availability

The datasets generated and analysed during the current study are available from the corresponding author on reasonable request.

## Abbreviations

BSA: Body surface area
C: Compliance
CD: Combined dysfunction
C_global_: Total systemic compliances (arterial and venous)
CI: Cardiac index
CO: Cardiac output
CVP: Central venous pressure
DBP: Diastolic blood pressure
DD: Diastolic dysfunction
EDP: End-diastolic pressure
EDPVR: End-diastolic pressure–volume relation
EDV: End-diastolic volume
EF: Ejection fraction
E_max_: Maximum heart chamber elastance
E_min_: Minimum heart chamber elastance
ESPVR: End-systolic pressure–volume relation
ESV: End-systolic volume
ET: Ejection time
HF: Heart failure
HR: Heart rate
LA: Left atrium
LV: Left ventricle
m_1_: Ascending steepness of the elastance of each heart chamber
m_2_: Descending steepness of the elastance of each heart chamber
MPI: Myocardial performance index
*p*: Percentage of impairment
P: Pressure
PAH: Pulmonary arterial hypertension
P_PuA_: Pulmonary arterial pressure
P_ra_: Right atrial pressure
P_rv_: Right ventricular pressure
PV: Pressure–volume
PVR: Pulmonary vascular resistance
Q_pv_: Pulmonary valve flow
Q_tv_: Tricuspid valve flow
R: Resistance
RA: Right atrium
RV: Right ventricle
SBP: Systolic blood pressure
SD: Systolic dysfunction
SV: Stroke volume
SVR: Systemic vascular resistance
τ_1_: Ascending elastance relative appearance in time within the heartbeat of each heart chamber
τ_2_: Descending elastance relative appearance in time within the heartbeat of each heart chamber
TCO: Atrioventricular closure opening time
t_onset_: Time-shift for the atrial contraction
V: Volume
VAD: Ventricular assist device
V_p__=__0_: Unstressed heart chamber volume
