# Supplementary material for: Understanding and recognition of the right ventricular function and dysfunction via a numerical study
Source: Sci Rep. 2021 Feb 12;11:3709. doi: 10.1038/s41598-021-82567-9 (PMC7881145; doi:10.1038/s41598-021-82567-9)
Supplement: Supplementary file 1 — Supplementary Information. [file 41598_2021_82567_MOESM1_ESM.pdf]

Understanding and recognition of the right ventricular function and dysfunction via a numerical study

Giulia Comunale[1*], Paolo Peruzzo[1], Biagio Castaldi[2], Renato Razzolini[3], Giovanni Di Salvo[2], Massimo A. Padalino[4], Francesca M. Susin[1].

[1] Cardiovascular Fluid Dynamics Laboratory HER, Department of Civil, Environmental and Architectural Engineering – University of Padova, Italy.

[2] Department of Women's and Children's Health, University of Padova Medical School, Italy.

[3] Cardiology Unit, Department of Cardiac, Thoracic and Vascular Sciences and Public Health, University of Padova Medical School, Italy.

[4] Paediatric and Congenital Cardiovascular Surgery Unit, Department of Cardio-thoracic and Vascular Sciences and Public Health, University of Padova Medical School, Italy.

Supplementary Information

Methods

Hemodynamic model

To simulate the blood circulation, we used the lumped parameters methodology. It allows to represent the vascular compartments of the body, i.e., any vascular segment that it is necessary to describe, by pressure and flow rate, and the heart. This methodology is widely used to simulate the whole cardiovascular system since the Windkessel model proposed by Otto Frank in 1899 which approximates the hemodynamics of the systemic circulation[11–13]. Pressures and flows are obtained by computing the Navier-Stokes and mass conservation equations averaged over the domain[14]. The vascular compartments are described by lumped parameters able to represent the geometrical and physical features. Resistances (R) represent the resistance to flow due to blood viscosity, compliances (C) reproduce the elastic properties of the vessels, and the inductances (L) reflect the flow inertia. By the combination of these parameters, any section of the vascular system can be considered. Particularly, supplementary Fig. S1 represents an example of a compartment, described by

$$\begin{cases} C\frac{dP_j}{dt} = Q_j - Q_{j+1} \\ L\frac{dQ_{j+1}}{dt} = P_j - P_{j+1} - RQ_{j+1} \end{cases} \tag{S1}$$

where (P_j, Q_j) and (P_{j+1}, Q_{j+1}) are the upstream and downstream pressures and flow rates, respectively. In this work, the great vessels were reproduced considering the compliance and resistance effects, and the vascular bed was represented by the resistance effect (supplementary Fig. S2). This holds for both the systemic and pulmonary circulations. This choice allows to reproduce the elastic properties and small dissipative effects of the arteries and veins, and the dissipation effects that characterize the small vessels of the vascular bed. Notice that preliminary tests showed that the adoption of a Windkessel model with more elements than those here included does not significantly refine the results of right ventricular dysfunction simulations. Finally, the heart valves were represented as an ideal diode associated to a

resistance. They open and close instantaneously and they allow the blood to flow through only when there is a positive pressure gradient across them, forcing a unidirectional flow. The equation is

$$Q = \begin{cases} \frac{P_j - P_{j+1}}{R}, & if\ P_j - P_{j+1} \geq 0 \\ 0, & otherwise. \end{cases} \tag{S2}$$

with P_j and P_{j+1} the pressure upstream and downstream, respectively, R the valve resistance and Q the flow rate.

Note that, the present model does not account for chamber interaction via septa[61–63].

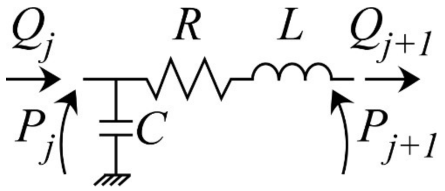

Figure S1. Example of lumped parameter compartment. *R*, resistance, *C*, compliance, *L*, inductance, (P_j, Q_j) and (P_{j+1}, Q_{j+1}) the upstream and downstream pressures and flow rates, respectively.

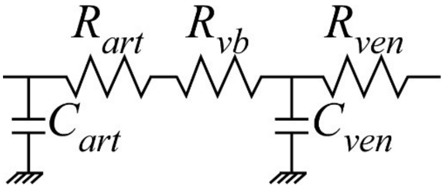

Figure S2. Lumped parameter compartment used in the present work to represent the systemic and pulmonary circulations. The arterial and venous sides are composed by a resistance and a compliance (R_{art}, C_{art}) and (R_{ven}, C_{ven}), respectively, whereas the vascular bed is represented by a resistance (R_{vb}).

Time-varying elastance

To simulate the right ventricular dysfunction, the right time-varying elastances were modified. Supplementary Fig. S3 shows the right time-varying elastance for the systolic, diastolic, and combined dysfunctions, resulted from the change of the parameters of Eq. (1), as described in Section Dysfunctional case. In the systolic dysfunction (Fig. S3a), $E_{max_{RV}}$, the maximum contraction force, decreases linearly with *p* increasing, from 0.45 mmHg/mL in the healthy condition to the minimum value of $E_{min_{RV}}$ for the

complete impairment ($E_{min_{RV}}$= 0.035 mmHg/mL). At the same time, the ejection time increases as well as the acceleration time, resulting in a delayed of the peak as the pathology worsens. On the contrary, in the diastolic dysfunction (Fig. S3b), $E_{max_{RV}}$ is rather constant and the systolic phase slightly varies with *p*, ranging between the 37% and the 40% of the heartbeat. However, an increase of ventricular stiffness and the decrease of the deceleration times are due to larger value of $E_{min_{RV}}$ and $m_{2_{RV}}$, respectively. Finally, in the combined dysfunction (Fig. S3c) the combination of the previous effects is visible.

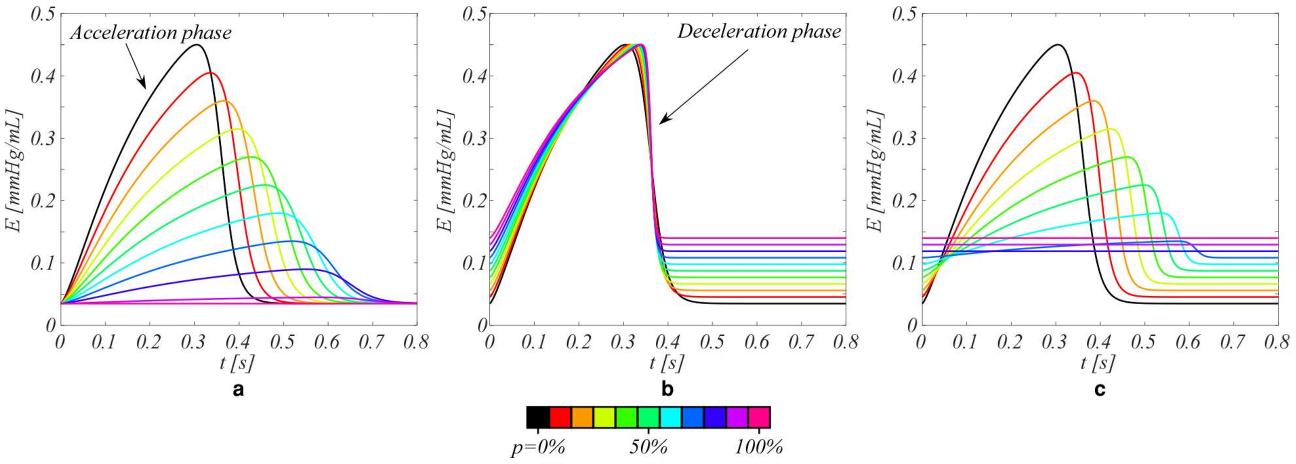

Figure S3. Right ventricular activation function. **(a)** systolic dysfunction, **(b)** diastolic dysfunction, and **(c)** combined systolic and diastolic dysfunction. Black line: healthy right ventricle (RV) (*p=0%*); coloured lines; the corresponded degree of RV impairment, *p*, from *10%* to *100%*.

Sensitivity analysis

A sensitivity analysis as in Mynard[19] was conducted to evaluate the sensitivity of the model. Input parameters were increased by 25%, one at a time, assessing the changes in the outputs. The sensitivity was computed considering the mean value of the outputs over one heartbeat as

$$S = \frac{mean(Y_1) - mean(Y)}{mean(Y)} \left(\frac{X}{X_1 - X} \right) \quad \text{(S6)}$$

where X and X_1 are the input at baseline and 25% increased value, respectively, and Y and Y_1 are the outputs with baseline and 25% increased inputs, respectively. Note that a positive value of S indicates that an increase in X determines an increase in Y, whereas, a negative value of S shows that an increase

in X causes a decrease in Y[19]. Particularly, we evaluated 16 inputs and 20 outputs of the model. Table S3 reports the results. The inputs are ordered considering the global influence, i.e., as the sum of $|S|$ for all the outputs analyzed, showing the most influential on the left. The outputs are ordered based on the overall sensitivity, computed as the median value of $|S|$ for all the inputs, with the most sensitive at the top. Supplementary Fig. S4 shows the outputs listed on the horizontal axis and inputs listed on the vertical axis. Note that in the figure, the outputs are ordered with the most sensitive on the left, and the inputs are ordered with the most influential at the bottom. The most influential parameters are those related to the heart chamber contractility. These parameters are known from the physiology to affect pressures and flows, however, interestingly, the contractility of LV is less influential than that of the other heart chambers. This may be due to the extended research attention posed on LV compared to the other heart chambers, suggesting that the left ventricular parameters are better estimated. Thus, the higher sensitivity of RA, RV, and LA parameters suggests that more attention needs to be posed when calibrating these heart chambers to derive pertinent values and be able to simulate real cases. The most sensitive outputs are those related to the systemic arterial side and the volumes, that are directly linked to the heart chamber contractility. Moreover, only few output variables are sensitive to the variation of inputs, and this is clearly visible from Supplementary Fig. S4 that shows a cluster of larger data points in the left side. The least sensitive outputs signals are the right ventricular and pulmonary venous pressures, and this could be related to the reservoir functionality of the high-compliance pulmonary circulation.

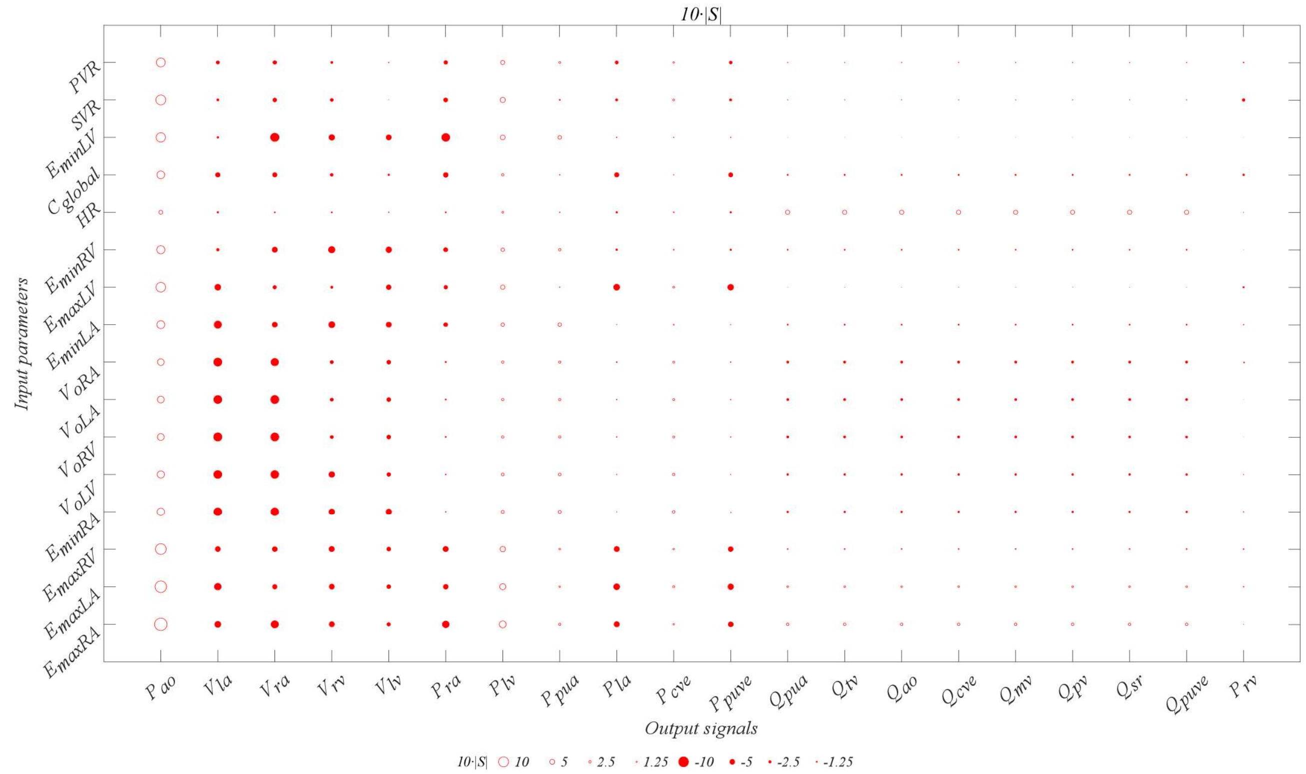

Figure S4. Graphical representation of the sensitivity of the outputs to the 25% variation of the inputs $\left(S = \frac{mean(Y_1)-mean(Y)}{mean(Y)}\left(\frac{X}{X_1-X}\right)\right)$. A larger symbol corresponds with higher sensitivity and filled/empty circles correspond to negative/positive S. Outputs are ordered according to the overall sensitivity (the most sensitive on the left) and inputs are ordered based on the global influence (the most influential at the bottom).

Tables

Table S1. Input parameters required to run the simulation: heart parameters, resistances and compliances of the circulation, and the initial values of the variables.

Heart parameters values				
	**LV**	**RV**	**LA**	**RA**
$\boldsymbol{E_{max}}$ $[mmHg/mL]$	2.8	0.45	0.13	0.09
$\boldsymbol{E_{min}}$ $[mmHg/mL]$	0.07	0.035	0.09	0.045
$\boldsymbol{V_{p=0}}$ $[mL]$	20	30	3	7
$\boldsymbol{\tau_1}$ $[s]$	0.269*T*	0.269*T*	0.110*T*	0.110*T*
$\boldsymbol{\tau_2}$ $[s]$	0.452*T*	0.452*T*	0.180*T*	0.180*T*
$\boldsymbol{m_1}$ $[-]$	1.32	1.32	1.99	1.99
$\boldsymbol{m_2}$ $[-]$	21.9	21.9	11.2	11.2
$\boldsymbol{t_{onset}}$ $[s]$	0	0	0.85*T*	0.85*T*
$\boldsymbol{R_{valve}}$ $[mmHg \cdot s/mL]$	0.01	0.01	0.005	0.005

Model parameters		***Initial values***	
$\boldsymbol{R_{systemic\,arteries}}$ $[mmHg \cdot s/mL]$	0.0448	$\boldsymbol{P_{systemic\,arteries}}$ $[mmHg]$	100
$\boldsymbol{R_{systemic\,vascular\,berd}}$ $[mmHg \cdot s/mL]$	0.824	$\boldsymbol{P_{systemic\,veins}}$ $[mmHg]$	4
$\boldsymbol{R_{systemic\,veins}}$ $[mmHg \cdot s/mL]$	0.0269	$\boldsymbol{P_{pulmonary\,arteries}}$ $[mmHg]$	15
$\boldsymbol{R_{pulmonary\,arteries}}$ $[mmHg \cdot s/mL]$	0.003	$\boldsymbol{P_{pulmonary\,veins}}$ $[mmHg]$	8
$\boldsymbol{R_{pulmonary\,vascular\,berd}}$ $[mmHg \cdot s/mL]$	0.0552	$\boldsymbol{V_{p=0_{lv}}}$ $[mL]$	149.6
$\boldsymbol{R_{pulmonary\,veins}}$ $[mmHg \cdot s/mL]$	0.0018	$\boldsymbol{V_{p=0_{rv}}}$ $[mL]$	189.2
$\boldsymbol{C_{systemic\,arteries}}$ $[mL/mmHg]$	0.983	$\boldsymbol{V_{p=0_{la}}}$ $[mL]$	71
$\boldsymbol{C_{systemic\,veins}}$ $[mL/mmHg]$	29.499	$\boldsymbol{V_{p=0_{ra}}}$ $[mL]$	67
$\boldsymbol{C_{pulmonary\,arteries}}$ $[mL/mmHg]$	6.7		
$\boldsymbol{C_{pulmonary\,veins}}$ $[mL/mmHg]$	15.8		

T is the heart period.

Table S2. Significant numerical values of pressures and flows computed for a subset of impairments *p* in the three considered types of dysfunction and compared to the healthy condition. Qualitative trends for the complete set are reported in Fig. 3.

	Healthy Condition	***Systolic Dysfunction (SD)***		***Diastolic Dysfunction (DD)***		***Combined Dysfunction (CD)***	
	p=0%	***p=50%***	***p=100%***	***p=50%***	***p=100%***	***p=50%***	***p=100%***
Right atrial pressure (Pra)							
RAP [mmHg]	4	5	7	6	8	7	9
Right ventricular pressure (Prv)							
Systolic [mmHg]	21	17	9	17	14	13	12
Diastolic [mmHg]	2	3	7	4	5	6	8
End-diastolic pressure [mmHg]	4	5	8	9	11	10	12
Pulmonary arterial pressure (Ppua)							
Systolic [mmHg]	18	15	8	15	13	12	10
Diastolic [mmHg]	11	10	7	9	8	9	8
Tricuspid valve flow (Qtv)							
E peak [mL/s]	364	328	-	470	492	340	-
A peak [mL/s]	333	444	508	379	391	421	498
Pulmonary valve flow (Qpv)							
Percentage of ejection time (ET) [%]	36	62	100	43	53	69	100

Table S3. Mean sensitivity (S) of output signals to the input parameters. The inputs are ordered based on the global influence (the most influential on the left), the outputs are ordered based on the overall sensitivity (the most sensitive at the top).

Output signals	Input parameters															
	$E_{max_{RA}}$	$E_{max_{LA}}$	$E_{max_{RV}}$	$E_{min_{RA}}$	$V_{p=0_{LV}}$	$V_{p=0_{RV}}$	$V_{p=0_{LA}}$	$V_{p=0_{RA}}$	$E_{min_{LA}}$	$E_{max_{LV}}$	$E_{min_{RV}}$	HR	C_{global}	$E_{min_{LV}}$	SVR	PVR
P_{ao}	1.251	1.154	1.077	0.750	0.724	0.685	0.681	0.672	0.798	0.963	0.817	0.408	0.778	0.939	0.995	0.892
V_{la}	-0.615	-0.679	-0.517	-0.796	-0.813	-0.838	-0.809	-0.815	-0.758	-0.600	-0.280	-0.170	-0.448	-0.180	-0.234	-0.327
V_{ra}	-0.737	-0.458	-0.493	-0.795	-0.811	-0.835	-0.837	-0.762	-0.521	-0.360	-0.532	-0.113	-0.447	-0.850	-0.389	-0.366
V_{rv}	-0.535	-0.521	-0.536	-0.575	-0.588	-0.324	-0.326	-0.331	-0.632	-0.254	-0.656	-0.120	-0.297	-0.569	-0.308	-0.228
V_{lv}	-0.355	-0.410	-0.409	-0.564	-0.382	-0.404	-0.406	-0.411	-0.533	-0.475	-0.583	-0.068	-0.179	-0.512	0.016	-0.061
P_{ra}	-0.674	-0.494	-0.532	-0.079	-0.101	-0.133	-0.136	-0.144	-0.415	-0.384	-0.428	-0.117	-0.479	-0.801	-0.417	-0.390
P_{lv}	0.692	0.604	0.541	0.315	0.291	0.256	0.252	0.244	0.360	0.438	0.367	0.187	0.239	0.481	0.521	0.425
P_{pua}	0.240	0.156	0.169	0.313	0.289	0.254	0.251	0.242	0.368	0.067	0.256	0.079	0.080	0.378	-0.121	0.200
P_{la}	-0.545	-0.613	-0.532	-0.026	-0.048	-0.081	-0.084	-0.091	0.022	-0.618	-0.185	-0.174	-0.460	-0.079	-0.240	-0.336
P_{cve}	0.153	0.214	0.159	0.277	0.254	0.219	0.215	0.207	0.106	0.194	0.107	0.109	0.061	-0.053	0.190	0.161
P_{puve}	-0.505	-0.574	-0.497	-0.009	-0.031	-0.064	-0.067	-0.075	0.040	-0.584	-0.163	-0.161	-0.433	-0.056	-0.234	-0.309
Q_{pua}	0.266	0.180	0.116	-0.172	-0.192	-0.224	-0.227	-0.234	-0.121	0.016	-0.104	0.431	-0.135	0.011	0.045	-0.042
Q_{tv}	0.265	0.180	0.116	-0.171	-0.192	-0.224	-0.227	-0.234	-0.122	0.016	-0.105	0.430	-0.135	0.009	0.043	-0.043
Q_{ao}	0.267	0.180	0.117	-0.171	-0.192	-0.223	-0.226	-0.234	-0.120	0.017	-0.104	0.431	-0.134	0.011	0.045	-0.042
Q_{cve}	0.267	0.180	0.117	-0.171	-0.192	-0.223	-0.227	-0.234	-0.120	0.017	-0.103	0.431	-0.134	0.011	0.045	-0.042
Q_{mv}	0.266	0.180	0.116	-0.170	-0.191	-0.223	-0.226	-0.233	-0.120	0.016	-0.103	0.430	-0.135	0.011	0.044	-0.043
Q_{pv}	0.267	0.181	0.117	-0.171	-0.192	-0.223	-0.226	-0.234	-0.120	0.017	-0.103	0.431	-0.134	0.011	0.045	-0.042
Q_{sr}	0.266	0.180	0.117	-0.171	-0.192	-0.224	-0.227	-0.234	-0.120	0.016	-0.104	0.431	-0.134	0.011	0.045	-0.042
Q_{puve}	0.267	0.181	0.118	-0.170	-0.191	-0.223	-0.226	-0.233	-0.119	0.017	-0.103	0.431	-0.133	0.012	0.045	-0.041
P_{rv}	-0.024	-0.080	-0.092	0.065	0.042	0.009	0.006	-0.002	0.075	-0.159	0.011	-0.024	-0.195	-0.007	-0.268	-0.089

P_{ao}, aortic (arterial vessel) pressure, V_{la}, LA volume, V_{ra}, RA volume, V_{rv}, RV volume, V_{lv}, LV volume, P_{ra}, RA pressure, P_{lv}, LV pressure, P_{pua}, PuA pressure, P_{la}, LA pressure, P_{cve}, CVe pressure, P_{puve}, PuVe pressure, Q_{pua}, PuA flow, Q_{tv}, tricuspid valve flow, Q_{ao}, aortic valve flow, Q_{cve}, CVe flow, Q_{mv}, mitral valve flow, Q_{pv}, pulmonary valve flow, Q_{sr}, systemic vascular bed flow, Q_{puve}, PuVe flow, and P_{rv}, RV pressure.

Additional references

61. Maksuti, E. *et al.* Cardiac remodeling in aortic and mitral valve disease: A simulation study with clinical validation. *J. Appl. Physiol.* **126**, 1377–1389 (2019).

62. Bozkurt, S. Mathematical modeling of cardiac function to evaluate clinical cases in adults and children. *PLoS One* **14**, 1–20 (2019).

63. Seemann, F. *et al.* Noninvasive Quantification of Pressure-Volume Loops From Brachial Pressure and Cardiovascular Magnetic Resonance. *Circ. Cardiovasc. Imaging* **12**, e008493 (2019).
